# Detecting Nasal Vowels in Speech Interfaces Based on Surface Electromyography

**DOI:** 10.1371/journal.pone.0127040

**Published:** 2015-06-12

**Authors:** João Freitas, António Teixeira, Samuel Silva, Catarina Oliveira, Miguel Sales Dias

**Affiliations:** 1 Microsoft Language Development Center, Microsoft Portugal, Lisboa, Portugal; 2 Department of Electronics Telecommunications & Informatics (DETI), University of Aveiro, Aveiro, Portugal; 3 Institute for Electronics and Telematics Engineering (IEETA), University de Aveiro, Aveiro, Portugal; 4 Health School, University of Aveiro, Aveiro, Portugal; 5 Instituto Universitário de Lisboa (ISCTE-IUL), ISTAR-IUL, Lisboa, Portugal; Duke University, UNITED STATES

## Abstract

Nasality is a very important characteristic of several languages, European Portuguese being one of them. This paper addresses the challenge of nasality detection in surface electromyography (EMG) based speech interfaces. We explore the existence of useful information about the velum movement and also assess if muscles deeper down in the face and neck region can be measured using surface electrodes, and the best electrode location to do so. The procedure we adopted uses Real-Time Magnetic Resonance Imaging (RT-MRI), collected from a set of speakers, providing a method to interpret EMG data. By ensuring compatible data recording conditions, and proper time alignment between the EMG and the RT-MRI data, we are able to accurately estimate the time when the velum moves and the type of movement when a nasal vowel occurs. The combination of these two sources revealed interesting and distinct characteristics in the EMG signal when a nasal vowel is uttered, which motivated a classification experiment. Overall results of this experiment provide evidence that it is possible to detect velum movement using sensors positioned below the ear, between mastoid process and the mandible, in the upper neck region. In a frame-based classification scenario, error rates as low as 32.5% for all speakers and 23.4% for the best speaker have been achieved, for nasal vowel detection. This outcome stands as an encouraging result, fostering the grounds for deeper exploration of the proposed approach as a promising route to the development of an EMG-based speech interface for languages with strong nasal characteristics.

## Introduction

Speech-based human-computer interfaces have reached high accuracy levels in controlled environments and are now commercially available. However, robust speech recognition and improved user experience, with this type of interface, remains a challenge [1] and an attractive research topic [2,3]. One of the reasons for this has to do with the fact that a conventional speech interface solely relies on the acoustic signal. Hence, this type of interface becomes inappropriate when used in the presence of environmental noise, such as in office settings, or when used in situations where privacy or confidentiality is required. For the same reason, speech-impaired persons, such as those who were subjected to a laryngectomy, are unable to use this type of interface. In this regard, Silent Speech Interfaces (SSI) can be viewed as a possible alternative since they allow for communication to occur in the absence of an acoustic signal. Although SSI are still at an early stage of development, the latest results have shown that they can be used to help tackle the issues raised by speech-based interfaces [4]. Surface Electromyography (EMG) is, among others, one of the approaches reported in literature that is suitable for implementing SSI, having achieved promising results [5,6].

A known challenge in SSI, including those based on surface EMG, is the detection of the nasality phenomenon in speech production, being unclear if information on nasality can be retrieved from the EMG signal. Nasality is an important characteristic of several languages, such as European Portuguese (EP) [7,8], which is the selected language for the experiments reported here. Additionally, no SSI exists for EP and, as shown before [9], nasality can negatively impact accuracy for this language. Given the particular relevance of nasality for EP [10,11], we have conducted an experiment that aims at improving on the current state-of-the-art in this area by determining the possibility of detecting nasal vowels in surface EMG-based speech interfaces, and consequently improving this type of interaction system. It is important to note that, as explained later, in more detail, the literature does not provide enough information regarding the possibility of detecting the nasality phenomenon, with surface EMG, and which sensor positions might be more adequate, to that purpose. Therefore, the goal of this first, exploratory stage is not to develop a complete, fully functional SSI system, based on EMG, considering nasality, but to conduct an exploratory study that informs future research. The main idea behind this experiment focuses on investigating two types of data containing information about the velum movement: (1) images collected using real time magnetic resonance imaging (RT-MRI) and (2) the myoelectric signal collected using surface EMG sensors. By combining these two sources, ensuring compatible recording conditions and proper time alignment, we are able to accurately estimate the time when the velum moves and the type of movement (i.e. ascending or descending), and infer the differences between nasal and oral vowels using surface EMG.

The use of surface electrodes, to target deeper muscles, presents several difficulties. It is not clear to what extent a surface electrode can detect the myoelectric signal, not only because of the depth at which the target muscles are located, but also due to signal propagation conditions in different tissues and associated noise. Also, the signal output of surface electrodes in the region of the face and neck will probably reflect a high level of cross talk, i.e. a mixture of signals from muscles that lie in the vicinity of, or superimposed on, the muscle fibers of interest. Therefore, this paper not only analyses the possibility of nasal vowel detection, using surface EMG, but also assesses if deeper muscles can be sensed using surface electrodes in the regions of the face and neck and the best electrode location to do so. Addressing these problems is an integral part of a challenging research agenda with the potential to impact speech, health and accessibility technologies by improving nasal vowel recognition in speech and in silent speech interfaces.

The remainder of this document is structured as follows: the following subsections present a brief description of relevant muscles associated with nasality in speech production and previous work focusing on EMG to measure the muscles associated with the movement of the soft palate; Section 2 describes the methodology used in this experiment, including the corpora description for both data collections and the methods used to extract nasality information from RT-MRI and to synchronize signal information; Section 3 presents the results for the exploratory analysis and classification experiment; Section 4 discusses the main outcomes and limitations of this study in light of their importance to advance the state-of-the-art and to support further research on the subject. Finally, conclusions and future work are presented in section 5.

### Background

**Fig 1:**
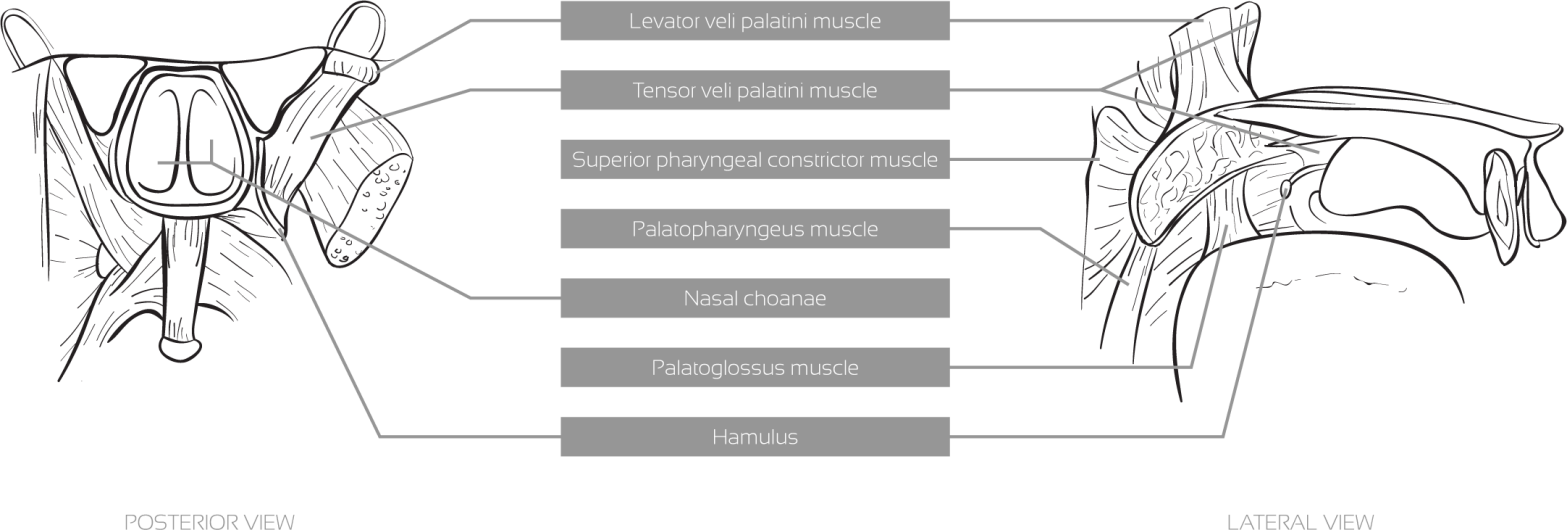
Muscles of the soft palate from posterior (left), and the side (right) view.

The production of a nasal vowel involves air flow through the oral and nasal cavities. This air passage for the nasal cavity is essentially controlled by the velum which, when lowered, allows for the velopharyngeal port to be open, enabling resonance in the nasal cavity, which causes the sound to be perceived as nasal. The production of oral sounds occurs when the velum is raised and the access to the nasal cavity is closed [12]. The process of moving the velum involves the following muscles [13–15], also depicted in Fig 1.

- *Levator veli palatini*: This muscle has its origin in the inferior surface of the apex of the petrous part of the temporal bone and its insertion in the superior surface of the palatine aponeurosis. Its main function is to elevate and retract the soft palate achieving velopharyngeal closure;
- *Musculus uvulae*: This muscle is embodied in the structure of the soft palate. In speech, it helps velopharyngeal closure by filling the space between the elevated velum and the posterior pharyngeal wall [16];
- *Superior pharyngeal constrictor*: Although this is a pharyngeal muscle, when it contracts it narrows the pharynx upper wall, which elevates the soft palate;
- *Tensor veli palatini*: This muscle tenses and spreads the soft palate and assists the *levator veli palatini* in elevating it. It also dilates the Eustachian tube. This muscle is innervated by means of the mandibular nerve of the V trigeminal, and not by the XI accessory nerve, as the remaining muscles of the soft palate. It is the only muscle of the soft palate that is innervated by a different nerve;
- *Palatoglossus*: Along with gravity, relaxation of the above-mentioned muscles and the *Palatopharyngeous*, this muscle is responsible for the lowering of the soft palate.

### Related Work

In previous studies, the application of EMG to measure the level of activity of these muscles has been performed by means of intramuscular electrodes [13,17] and surface electrodes positioned directly on the oral surface of the soft palate [18,19]. Our work differs from the cited papers, since none of them uses surface electrodes placed in the face and neck regions, a significantly less invasive approach and quite more realistic and representative of the SSI case scenarios. Also, although intramuscular electrodes may offer more reliable myoelectric signals, they also require considerable medical skills and, for that reason, intramuscular electrodes were discarded for this study.

To the best of our knowledge, no literature exists in terms of detecting the muscles involved in the velopharyngeal function with surface EMG electrodes placed on the face and neck. Previous studies, in the lumbar spine region, have shown that, if proper electrode positioning is considered, a representation of deeper muscles can be acquired [20], thus raising a question that is currently unanswered: Is surface EMG, positioned in the face and neck regions, able to detect activity of the muscles related to nasal port opening/closing and, consequently, detect the nasality phenomena? Another related question that can be raised is how we can ascertain, with some confidence, that the signal we are retrieving is, in fact, a myoelectric signal mostly generated by the velum movement and not by spurious movements caused by neighboring muscles, unrelated to the velopharyngeal function.

## Methodology

All human participants have provided informed written consent. The experiments reported in this study have been evaluated and approved by the ethics committee of the University Institute of Lisbon (ISCTE-IUL) regulated by dispatch n°7095/2011.

For determining the possibility of detecting nasal vowels using surface EMG we needed to know when the velum was moving, to avoid misinterpreting signals coming from other muscles, artifacts and noise, as signals coming from the target muscles. To overcome this problem we took advantage of a previous data collection based on RT-MRI [21], which provides an excellent method to estimate when the velum was moving and interpret EMG data accordingly.

Recent advances in MRI technology allow real-time visualization of the vocal tract with an acceptable spatial and temporal resolution. This technology enabled access to real time images with relevant articulatory information for our study, including velum raising and lowering. In order to align both signals, audio recordings were performed in both data collections for the same set of speakers. It is important to notice that EMG and RT-MRI data cannot be collected together, due to hardware restrictions, so the best option was to collect the same corpus for the same set of speakers, at different times, reading the same prompts in EMG and RT-MRI.

Both corpora (EMG and RT-MRI velum information) have been made available according to the journal’s data policy and can be accessed in http://sweet.ua.pt/sss/resources.

### Corpora

The two corpora collected in this study (RT-MRI and EMG) share the same prompts. The set of prompts is composed of several nonsense words containing five EP nasal vowels (using the Speech Assessment Methods Phonetic Alphabet (SAMPA) [6~, e~, i~, o~, u~]) isolated and in word-initial, word-internal and word-final context (e.g. ampa [6~p6], pampa [p6~p6], pam [p6~]). The nasal vowels were flanked by the bilabial stop or the labiodental fricative. For comparison purposes, the set of prompts also includes oral vowels, both isolated and in context. In the EMG data collection a total of 90 utterances per speaker were recorded. A more detailed description of the RT-MRI corpus can be found in [21]. In the EMG data collection, we have also recorded three silence prompts (i.e. prompts where the speaker does not speak or makes any kind of movement) to further validate the system and the acquired EMG signal.

For validation purposes, to support the assessment of aspects such as reproducibility, we have also recorded four additional EMG sessions, for one random speaker, with the same prompts.

#### Speakers

The three speakers participating in this study were all female, native speakers of EP, with the following ages: 33, 22 and 22 years. No history of hearing or speech disorders is known for any of them. The first speaker is a professor in the area of Phonetics and the remaining speakers are students in the area of Speech Therapy. All speakers have provided written and informed consent for the data collections.

Regarding the number of speakers considered, and how they might affect the results of this exploratory study, please bear in mind the following aspects. The aim of this work does not include proposing a finished system, but to explore the possibility of developing such system. It should be noted that the required resources, to accomplish this kind of complex studies, are very expensive, and a large data collection is strongly unadvisable without an adequate set of exploratory studies to assert its feasibility and applicability. Additionally, the literature, given the innovative nature of the presented work, does not provide enough information to allow skipping these initial steps. Therefore, we chose the number of speakers based on the data available from an existing RT-MRI study that suited our needs.

### RT-RMI Data

The RT-MRI data collection was previously conducted at IBILI/Coimbra for nasal production studies. Images were acquired in the midsagittal and coronal oblique planes of the vocal tract using an Ultra-Fast RF-spoiled Gradient Echo (GE) pulse sequence and yielding a frame rate of 14 frames/second. Each recorded sequence contained 75 images. Additional information concerning the image acquisition protocol can be found in [22].

The audio was recorded simultaneously with the real-time images, inside the scanner, at a sampling rate of 16000Hz, using a fiber optic microphone. For synchronization purposes a TTL pulse was generated from the RT-MRI scanner [21]. Currently, the corpus contains only three speakers due to costs per recording session and availability of the technology involved.

### Extraction of information on nasal port from RT-MRI data

For the mid-sagittal RT-MRI sequences of the vocal tract, since the main interest was to interpret velum position/movement from the sagittal RT-MRI sequences, instead of measuring distances (e.g. from velum tip to the posterior pharyngeal wall), we opted for a method based on the area variation between the velum and pharynx, which is closely related to velum position.

**Fig 2:**
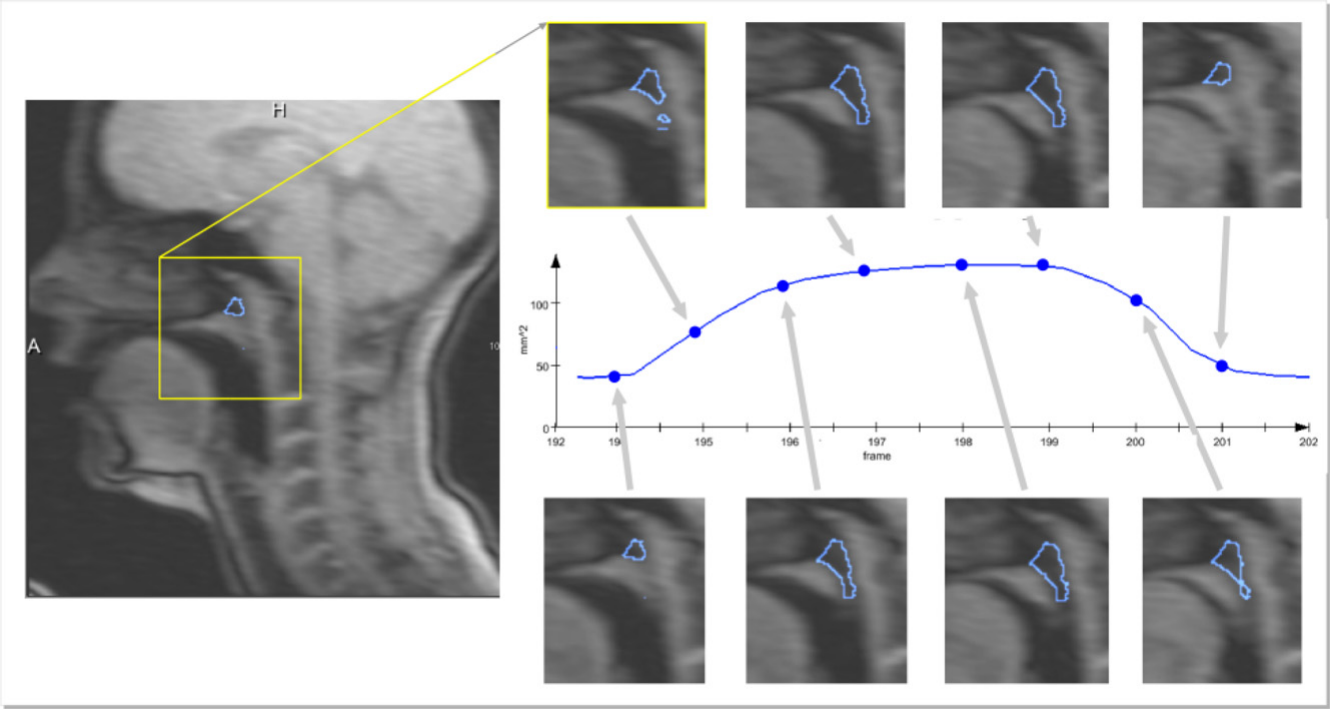
Mid-sagittal RT-MRI images of the vocal tract for several velum positions, over time, showing evolution from a raised velum, to a lowered velum and back to initial conditions. The presented curve, used for analysis, was derived from the images.

An image with the velum fully lowered was used to define a region of interest (ROI). Then, a region growing algorithm was applied with a seed defined in a hypo-intense pixel inside the ROI. This ROI is roughly positioned between the open velum and the back of the vocal tract. The main purpose is that the velum will move over that region when closing. Since this first ROI could be defined as enclosing a larger region, even including a part of the velum (which will not influence the process), it is only important that the seed is placed in a dark (hypo-intense) pixel inside it, in order to exclude the most of the velum from the region growing when it is positioned inside the ROI. Since there is spatial coherence between the images in each sequence, by defining a seed neighborhood including time, a single seed is typically enough to propagate the region growing inside the ROI over all image frames. Fig 2 presents the contours of the segmented region over different image frames encompassing velum lowering and rising. For representation purposes, in order not to occlude the image beneath, only the contour of the segmented region is presented. Processing is always performed over the pixels enclosed in the depicted region. Notice that the blue boundaries presented in the images depict the result of the region growing inside the defined ROI (which just limits the growth) and not the ROI itself. The number of hypo-intense pixels (corresponding to an area) inside the ROI decreases when the velum closes and increases when the velum opens. Therefore, a closed velum corresponds to area minima while an open velum corresponds to local area maxima, which allows detecting the frames where the velum is open. Since for all image sequences there was no informant movement, the ROI has to be set only once for each informant, and can then be reused throughout all the processed sagittal real-time sequences. After ROI definition (around one minute and reusable throughout all image sequences from the same speaker), setting a seed, revising the results and storing the data took one minute per image sequence.

**Fig 3:**
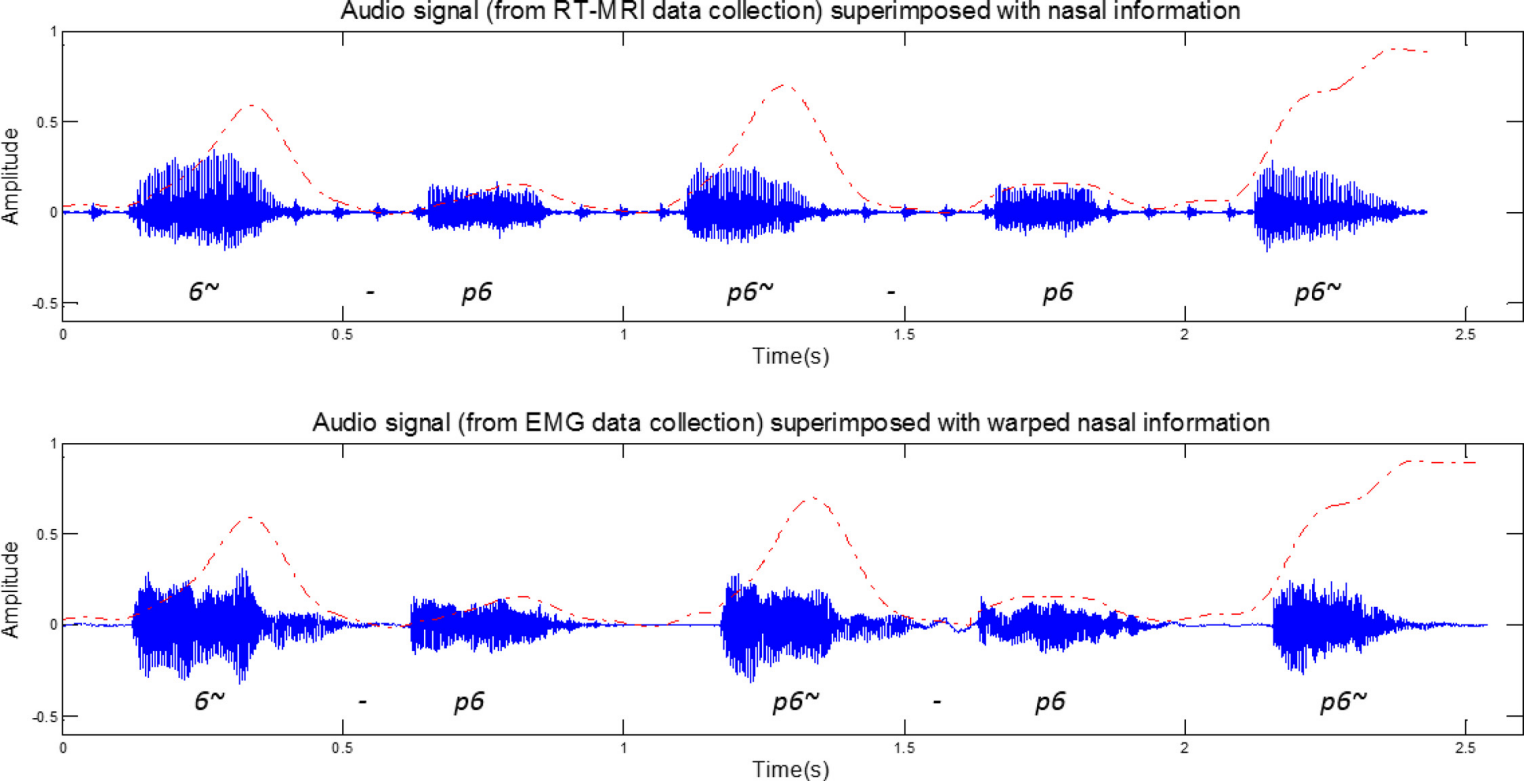
Exemplification of the warped signal representing the nasal information extracted from RT-MRI (dashed line) superimposed on the speech recorded during the corresponding RT-MRI and EMG acquisition, for the sentence [6~p6, p6~p6, p6~].

These images allowed deriving a signal, over time that describes the velum movement (also shown in Fig 2 and depicted as dashed line in Fig 3). As can be observed, minima correspond to a closed velopharingeal port (oral sound) and maxima to an open port (nasal sound).

Concerning the coronal-oblique image sequences, exploring the spatial coherence between image frames, a segmentation method based on region growing (with a neighborhood defined including time) was used. The user had to place a few seeds inside the oral cavity and inside the nasal cavity (typically four or five seeds in total). This process of segmenting the oral and nasal cavities, marking the seeds where needed, and obtaining/inspecting the oral and nasal cavities segmentations, by going through the image sequence, took around one minute per sequence. The area data for the nasal cavity was automatically computed and the resulting variation curve depicts the velum movement with the maxima and minima (zero) corresponding to an open and closed velopharyngeal port respectively. Additional details concerning the segmentation of the oblique real-time images can be found in [21].

**Fig 4:**
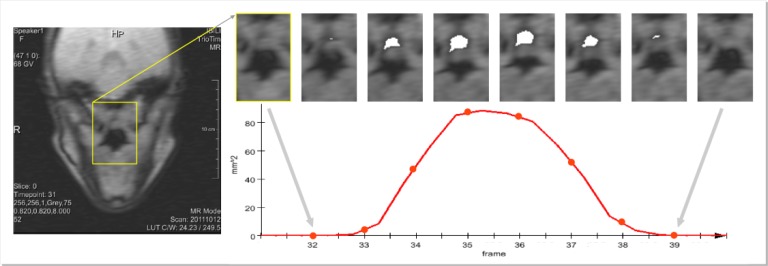
Coronal-oblique RT-MRI images depicting the nasal cavity (in white), over time, and the curve derived for analysis purposes.

Fig 4 presents different image frames showing the nasal cavity (depicted in white), encompassing lowering and rising of the velum and showing the derived curve.

### Surface EMG Data Collection

For this data collection the same speakers from the RT-MRI recordings were involved. For each speaker, the corpus was recorded completely in a single session, and the sensors were never removed during the recordings. Before placing the sensors, the sensor location was first cleaned with alcohol. While uttering the prompts an assistant asked the speaker to perform no other movement, besides the one associated with speech production, particularly any kind of neck movement. To minimize spurious muscle movements the speaker was allowed to rest between prompts, however, although the prompts had a short duration, we cannot guarantee the complete absence of innate movements such as breathing. The recordings took place in an isolated, quiet room. The assistant was responsible for pushing the record button and also for stopping the recording, to avoid unwanted muscle activity. The prompts were presented to the speaker in a random order and were selected based on the already existent RT-MRI corpus [21]. In this data collection two signals were acquired: myoelectric and audio. For synchronization purposes, after starting the recording, a marker was generated in both signals. In the sections below surface EMG and audio acquisition setups are described.

#### Surface EMG setup

The same acquisition system from Plux [23] was used to collect all EMG data. We used 5 pairs of EMG surface electrodes connected to a device that communicates with a computer via Bluetooth. The sensors were attached to the skin using single-use 2.5cm diameter clear plastic self-adhesive surfaces with approximately 2cm spacing between the electrodes’ center. One of the difficulties found, while preparing this study, was that no specific background literature in speech science exists to inform on the best sensor position to detect the muscles referred in section 0. Hence, based on anatomy and physiology literature (for example [14]) and preliminary trials, we determined sensor placement to cover, as much as possible, the positions that, most likely, are the best for detecting the targeted muscles. To measure the myoelectric activity we used both a bipolar and monopolar surface electrode configuration. In the monopolar configuration, instead of having both electrodes placed directly on the articulatory muscles (as in the bipolar configuration), one of the electrodes is used as a reference (i.e. located in a place with low or negligible muscle activity). In both configurations the result will be the amplified difference between the pair of electrodes.

**Fig 5:**
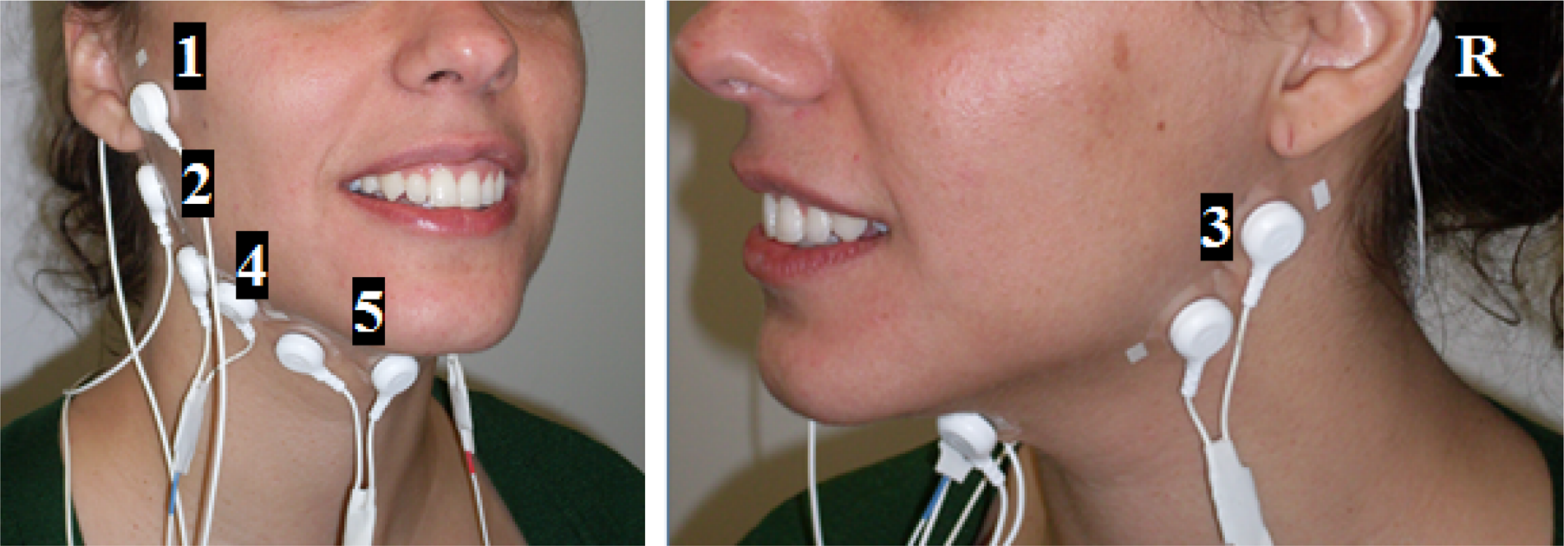
EMG electrodes positioning and the respective channels (1 to 5) plus the reference electrode (R). EMG 1 and 2 use unipolar configurations and EMG 3, 4 and 5 use bipolar configurations.

As depicted in Fig 5, the 5 sensors pairs were positioned in the following locations:

- EMG 1 was placed in the area superior to the mandibular notch, superficial to the mandibular fossa;
- EMG 2 was placed in the area inferior to the ear between the mastoid process and the mandible angle, on the right side of the face using a monopolar configuration;
- EMG 3 was also placed in the same position as EMG2 but used a bipolar configuration and was placed on the left side of the face;
- EMG 4 was placed in the superior neck area, beneath the mandibular corpus, at an equal distance from the mandible angle (EMG 2) and the mandible mental prominences (EMG 5);
- EMG 5 was placed in the superior neck area, beneath the mandible mental prominences.

The reference electrodes (EMG R) were placed in the mastoid portion of the temporal bone and in the cervical vertebrae. Considering the sensors’ location, they were expected to acquire unwanted myoelectric signals due to the superposition of the muscles in these areas, such as the jaw muscles. However, in spite of the muscles of the velum being remote from this peripheral region, we expected to be able to select a sensor location that enabled us to identify and classify the targeted muscle signal with success.

The technical specifications of the acquisition system [23] include snaps with a diameter of 1.46cm and 0.62cm of height, a voltage range that goes from 0.0V to 5.0V and a voltage gain of 1000. The recording signal was sampled at 600Hz and 12 bit samples were used. For system validation, we have conducted several preliminary tests on larger superficial muscles.

#### Audio system setup

The audio recordings were performed using a laptop integrated dual-microphone array using a sample rate of 8000Hz, 16 bits per sample and a single audio channel. Since the audio quality was not a requirement in this collection we opted for this solution instead of a headset microphone, which could cause interference with the EMG signal.

### Signal Synchronization

In order to address the nasal vowel detection problem we needed to synchronize the EMG and RT-MRI signals. Both the EMG and the RT-MRI datasets were aligned with their corresponding audio recordings. Next, we resampled the audio recordings to 12000Hz and applied Dynamic Time Warping (DTW) [24]to find the optimal match between corresponding audio recordings in both datasets. Based on the DTW result we mapped the information extracted from the RT-MRI to the EMG time axis, establishing the required correspondence between the EMG and the RT-MRI information, as depicted in Fig 3. In order to validate the alignment, we annotated the audio *corpora* of speaker 1 and compared the beginning of each word in the warped RT-MRI audio signal with the EMG audio signal. The relative position of the nasality signal of the EMG audio signal was found to be very similar to the one observed in the RT-MRI audio signal, which was an indication of good synchronization.

**Fig 6:**
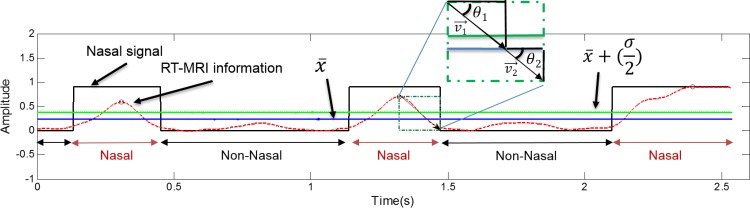
Exemplification of the EMG signal segmentation into nasal and non-nasal zones based on the information extracted from the RT-RMI (dashed red line). The square wave depicted with a black line represents the velum information split into two classes where 0 stands for non-nasal and 1 for nasal. The blue line is the average of the RT-MRI information (after normalization) and the green line is the average plus half of the standard deviation.

Based on the information extracted from the RT-MRI signal (after signal alignment), we were able to segment the EMG signal into nasal and non-nasal zones, using the zone boundary information to identify which parts of the EMG signal were nasal or non-nasal, as depicted in Fig 6. Considering a normalized RT-MRI signal *x*, we determined that *x(n)* ≥ $\bar{x}$ + (σ / 2) was nasal and *x(n)* < $\bar{x}$ + (σ / 2) was non-nasal, based on an empirical analysis that considered the signals from all users. However, in order to include the whole transitional part of the signal (i.e. lowering and raising of the velum) in the nasal zones, we used the angle between the nearest peak and the points where the *x(n)* = $\bar{x}$ to calculate the nasal zone boundaries. This was done after testing different methods and their ability to cope with signal variability among speakers.

As detailed in Fig 6, if we picture two right triangles formed between the peak and *x(n) =* 0, by knowing angle θ_1_ (between the peak and the *yy* axis), the opposing *cathetus* of θ_2_ and assuming that θ_1_ = θ_2_, we were able to determine the magnitude of *v*_2_ and set a zone boundary that included either the lowering or the raising part of the signal. In one of the speakers there were a few cases where the velum remains open for longer than expected. For these situations different peaks were used for the initial and end boundary of a nasal zone.

Regarding the different speaker postures during acquisition, between the RT-MRI and EMG data, and how these could influence the matching of both datasets, several studies have been presented in the literature concerning the differences, in vocal tract configuration, between sitting/upright and supine acquisitions of articulatory data. In general, mild differences in vocal tract shape and articulator position, due to body posture, were reported by several authors [25–28]. These mostly refer to an overall tendency of the articulators to deform according to gravity, resulting in more retracted positions of the tongue and, to a lesser extent, of the lips and lower end of the uvula in the supine acquisitions. To which extent this effect is observed varies between speakers [26]. None of these studies specifically addressed the velum or nasality, but analysis of vocal tract outlines, when they contemplate the velum [26], did not seem to show any major difference. Considering the acoustic properties of speech, one important finding was that no significant differences have been found between the two body postures, during acquisition [27,29]. Furthermore, the acquisition of running speech, as shown by Tiede et al. [25] and Engwall [29], minimizes vocal tract shape and articulator position differences occurring between upright and supine positions.

Concentrating on the velopharyngeal mechanism, Perry [30] studied its configuration for upright and supine positions during speech production, and concluded that no significant difference was present regarding velar length, velar height and *levator* muscle length.

Concerning muscle activity, Moon et al. [31] present a study where the activity of the *levator veli palatini* and *palatoglossus* muscles for upright and supine speaker postures was assessed using electromyography. The activation levels were smaller, for the supine position, during the closing movement (with gravity working in the same direction), but no timing differences were reported.

Therefore, considering the used methodology and the knowledge available in the literature, the different postures do not seem to be a major differencing factor between the two datasets that would preclude their matching through the acoustic signal. Furthermore, no evidence exists that muscle activity is relevantly affected by speaker posture. For both postures, apart from different activation levels, similar muscular configurations and activity seem to be observed, supporting the assumption that, after aligning both datasets, muscle activity relating to the velopharyngeal mechanism should also be aligned between both signals. This allows inferring muscle activity intervals from velum movement extracted from the MRI images.

### Data processing and analysis methods

**Fig 7:**
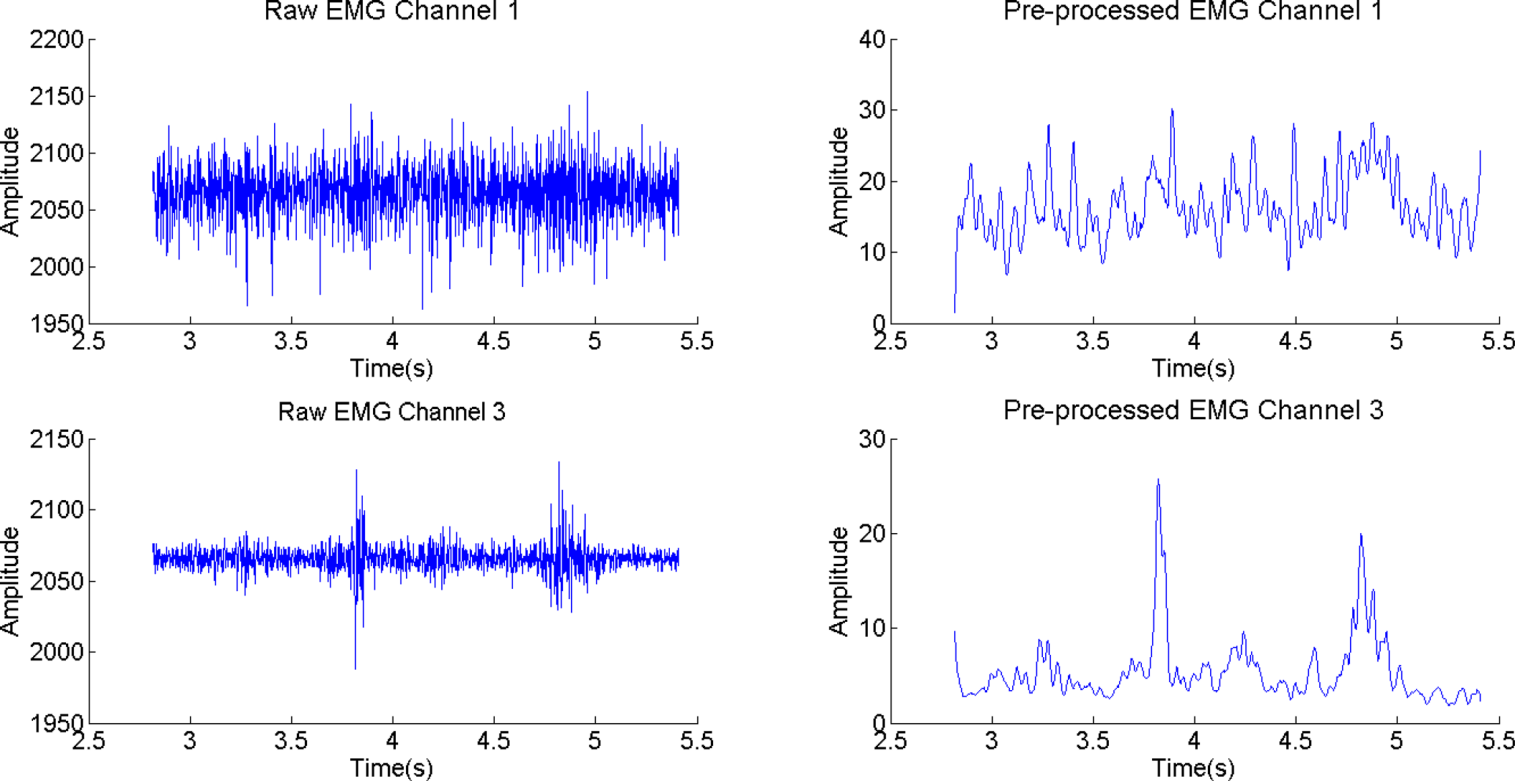
Raw EMG signal and pre-processed EMG signal of channel 1 (top) and 3 (bottom) for the sentence [6~p6, p6~p6, p6~] from speaker 1. The pre-processed signal has been normalized and filtered using a 12-point moving average filter.

In order to facilitate the analysis, we first pre-processed the EMG signal by normalizing it and applying a 12-point moving average filter with zero-phase distortion to the absolute value of the normalized EMG signal. An example of this pre-processing is depicted in Fig 7.

In the signal analysis, to measure the dependence between the MRI information and the EMG signal we have used the mutual information concept. The mutual information [32,33], also referred to as transinformation, was derived from the bases of information theory, and is given by:

$$I\left(X;Y\right)=\;\sum_{y€Y}\sum_{x€X}p\left(x,\;y\right)\;log\left(\frac{p\left(x,\;y\right)}{p\left(x\right)\;p\left(y\right)}\right)$$ (1)

where *p(x*,*y)* is the joint probability distribution function of *X* and *Y*, and *p(x)* and *p(y)* are the marginal probability distribution functions of *X* and *Y* respectively. This measures the common dependence of the two variables, providing the difference of information of one signal given the other. In this study we used a normalized mutual information [34] measure to analyze the relation between the signals.

To estimate classifier performance we have split the EMG signal into 100ms frames with a frame shift of 20ms. Afterwards, we applied a 10-fold cross-validation technique to the whole set of frames from the 3 speakers to split the data into training and validation subsets.

From each frame we extracted 9 first order temporal features similar to the ones used by Hudgins et al. [35]. Our feature vector was then composed of mean, absolute mean, standard deviation, maximum, minimum, kurtosis, energy, zero-crossing rate and mean absolute slope. Both feature set and frame sizes were determined empirically after several experiments. For classification we used Support Vector Machines (SVM) with a Gaussian Radial Basis Function.

To confirm the existence of significant differences among the results of the EMG channels, we used an Analysis of Variance (ANOVA) of the error rate, using SPSS (SPSS 19.0 –SPSS Inc., Chicago, IL, USA) and R [36,37].

## Results

In this section the results of the analysis combining the EMG signal with the information extracted from the RT-MRI signal, two classification experiments, and a reproducibility assessment are presented. In the first classification experiment, the EMG signal was divided into frames and each frame was classified as being nasal or non-nasal. The second experiment also divides the EMG signal into frames, but the classification was made by nasal and non-nasal zones, whose limits were known a priori based on the information extracted from the RT-MRI. The final part of this section addresses the problem of EMG signal variability across recording sessions.

For the signal and statistical analysis of the EMG signal we considered 43 observations per speaker covering all EP nasal vowels, each containing the nasal vowel in several word positions (initial, internal and final) and flanked by [p]. We have also used, for visual analysis only, 4 observations per speaker containing isolated EP nasal vowels ([6~, e~, i~, o~, u~]).

### Exploratory Visual Analysis

**Fig 8:**
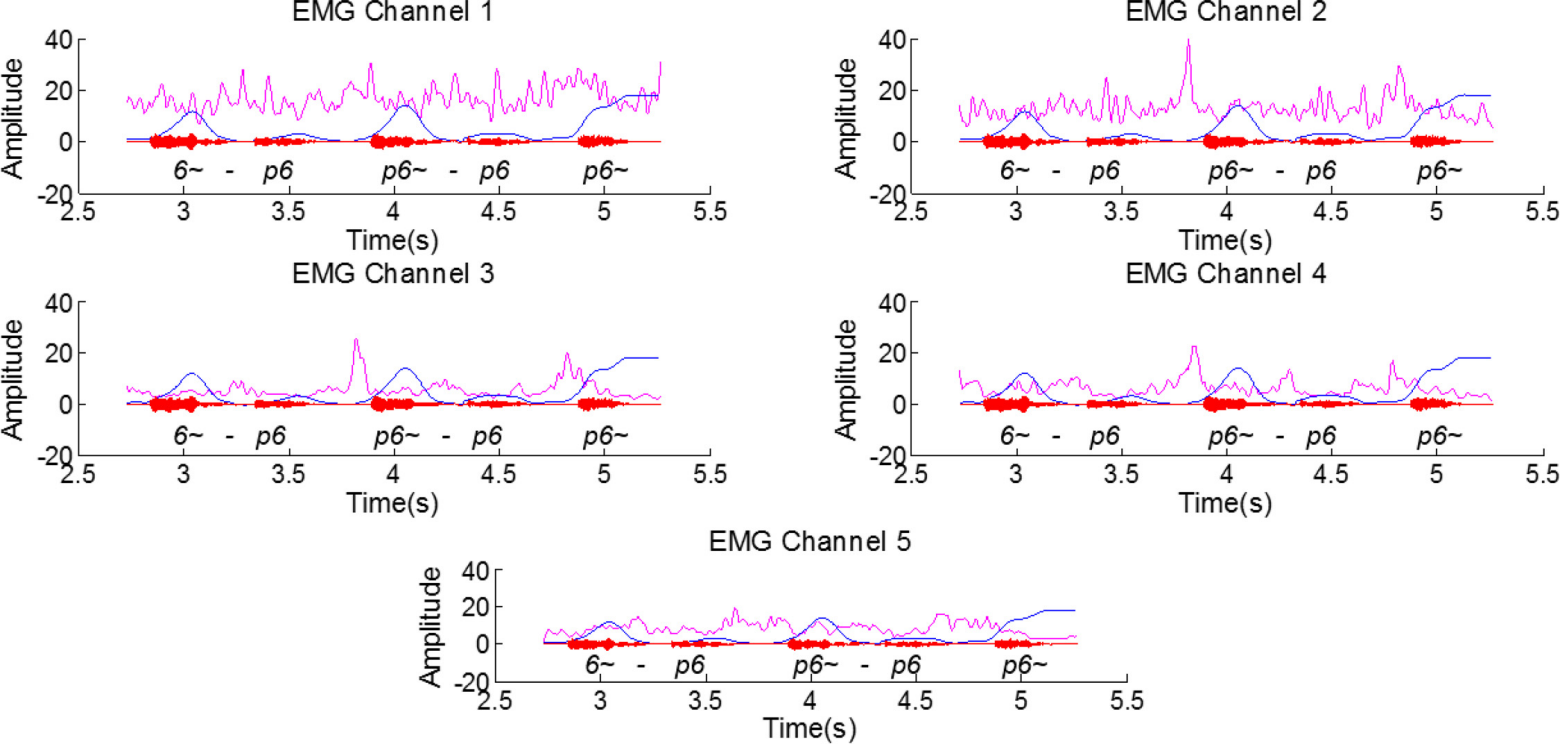
Filtered EMG signal (12-point moving average filter) for the several channels (pink), the aligned RT-MRI information (blue) and the respective audio signal for the sentence [6~p6, p6~p6, p6~] from speaker 1. An amplitude gain was applied to the RT-MRI information and to the EMG for better visualization of the superimposed signals.

After extracting the required information from the RT-MRI images and aligning it with the EMG signal we visually explored possible relations between the signals. The EMG signal, for all channels, after pre-processing, along with the data derived from the RT-MRI, aligned as described in the previous section, are depicted in Fig 8. Based on a visual analysis, it is worth noticing that several peaks anticipate the nasal sound, especially in channels 2, 3 and 4. These peaks are most accentuated for the middle and final word position.

**Fig 9:**
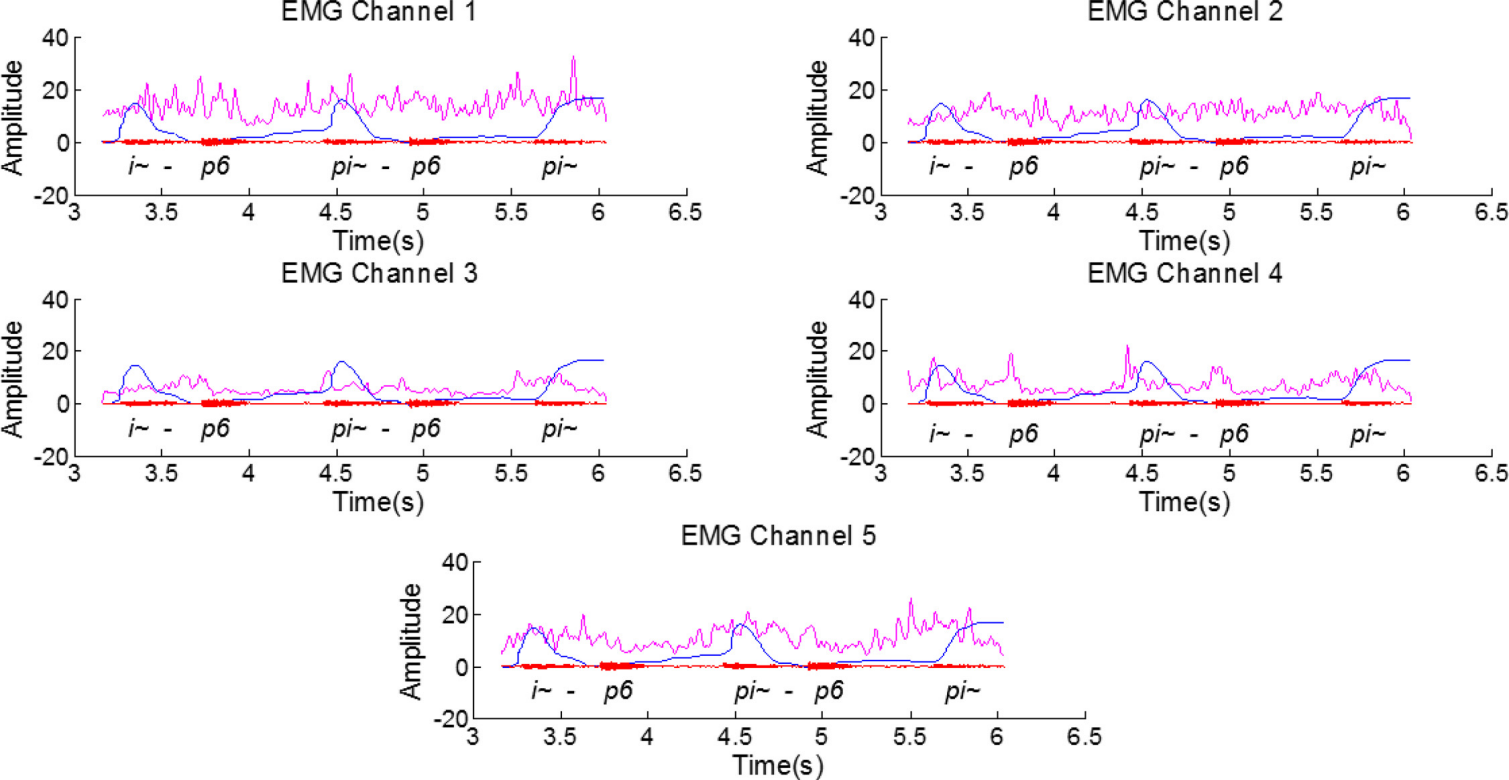
Filtered EMG signal (12-point moving average filter) for the several channels (pink), the aligned RT-MRI information (blue) and the respective audio signal for the sentence [i~p6, i~p6, pi~] from speaker 1. An amplitude gain was applied to the RT-MRI information and to the EMG for better visualization of the superimposed signals.

By using surface electrodes, the risk of acquiring myoelectric signal superposition is relatively high, particularly from muscles related with the movement of the lower jaw and the tongue, given the electrodes’ position. However, if we analyze an example of a close vowel such as [i~], for which the movement of the jaw is less prominent, the peaks found in the signal still anticipate the RT-MRI velar information for channels 3 and 4, as depicted in Fig 9. Channel 5 also exhibits a more active behavior in this case, which might be caused by its position near the tongue muscles and the tongue movement associated with the articulation of the [i~] vowel.

**Fig 10:**
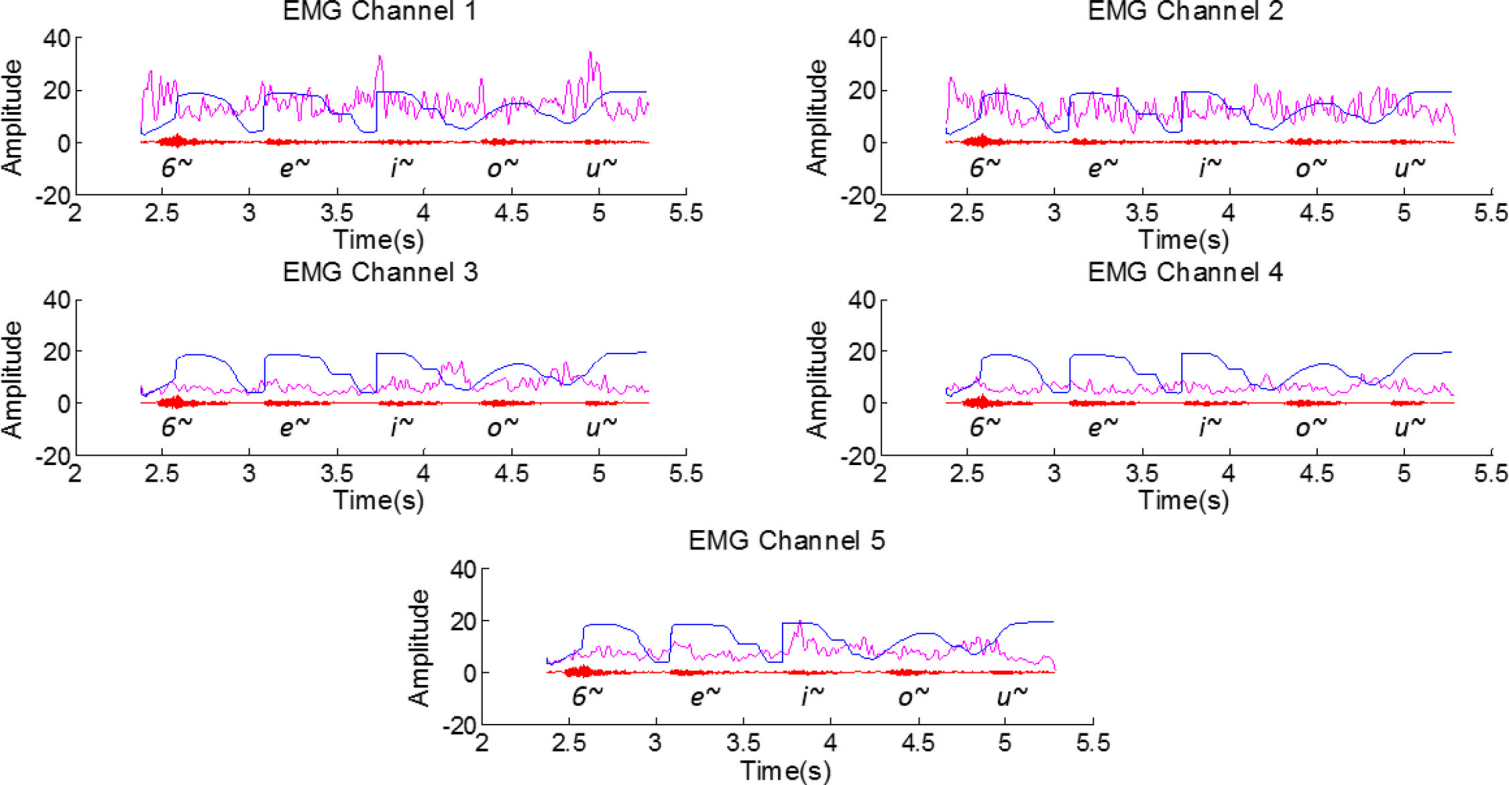
Portuguese vowels in an isolated context (Pre-processed EMG signal for all EMG channels (pink), the aligned RT-MRI information (blue) and the respective audio signal (red) for [6~, e~, i~, o~, u~]). An amplitude gain was applied to the RT-MRI information and to the EMG for better visualization of the superimposed signals.

Fig 10 shows the audio, RT-MRI and the EMG signal for an utterance that contains isolated EP nasal vowels in the following order: [6~, e~, i~, o~, u~]. These particular utterances are relevant since, in this case, minimal movement of the external articulators such as the lower jaw is required. If the same analysis is considered for isolated nasal vowels of the same speaker, EMG Channel 1 signal exhibits a clearer signal, apparently with less muscle crosstalk and peaks can be noticed before the nasal vowels. For the remaining channels there is not a clear relation with all the vowels, although signal amplitude variations can be noticed in the last three vowels for Channels 3 and 5.

**Fig 11:**
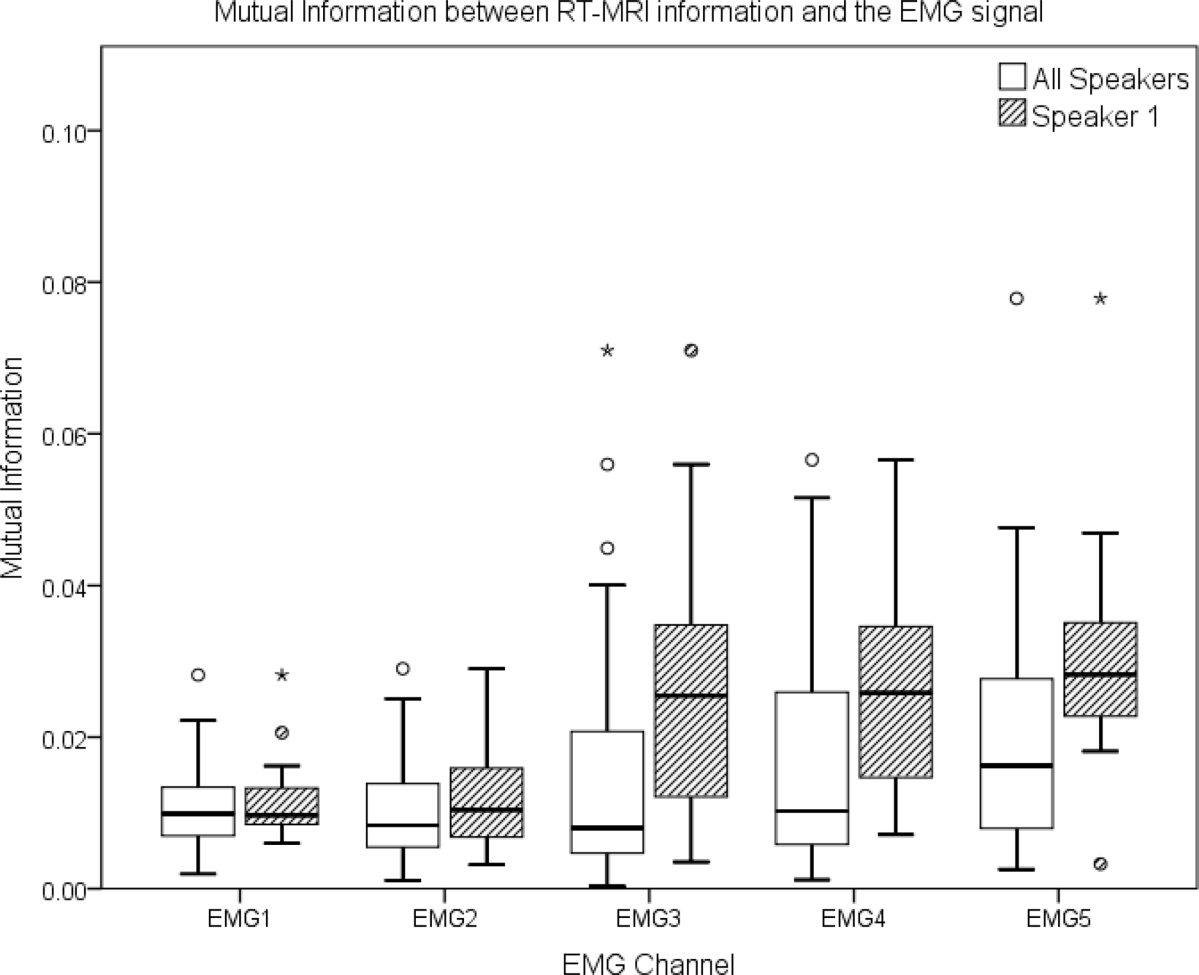
Boxplot of the mutual information in the nasal zones between the RT-MRI information and the EMG signal of all speakers and for a single speaker.

To confirm our first impressions, gathered from the analysis presented above, we have also conducted a quantitative analysis, investigating the existence of mutual information between the EMG signal and the information extracted from the RT-MRI signal. This measure of mutual dependence between the nasal zones of the RT-MRI signal and the EMG signal is depicted in Fig 11, allowing us to investigate the relation between the signals from another viewpoint. When conducting this analysis, considering the nasal zones for all speakers simultaneously, the mutual information values for channels 3, 4 and 5 were slightly higher, as depicted by the boxplots presented on Fig 11. When considering each speaker individually, we found that, at least for one speaker, the best results can be found for channels 3, 4 and 5 as well. Also, for the same speaker, we noticed that the amount of mutual information for non-nasal zones was close to zero.

Other situations, such as the relation between the RT-MRI signal and the non-nasal zones of the EMG signal and the relation between the RT-MRI signal and the whole EMG signal, were also analyzed but no relevant information was found. Also, other measures such as the Pearson’s product-moment correlation coefficient, which measures the degree of linear dependence between two signals, were also employed, but magnitudes below 0.1 were found, indicating a very weak linear relationship between the signals.

The fact that all seemed to point to the presence of differences between the two classes (nasal and non-nasal) motivated an exploratory classification experiment based on SVM [38], which has shown to yield acceptable performance in other applications, even when trained with small data sets. The results of this experiment are presented in what follows.

### Frame-based Nasality Classification

In a real use situation the information about the nasal and non-nasal zones, extracted from the RT-MRI signal, is not available. Thus, in order to complement our study, and because one of our goals is to have a nasality feature detector, we have conducted an experiment where we split the EMG signal into frames and classify them as one of two classes: nasal or non-nasal. Relevant statistics and distribution of the used dataset are described in Table 1.

**Table 1:** Class distribution for all speakers for a single EMG channel by zones and frames (nasal and non-nasal).

	**Speaker 1**	**Speaker 2**	**Speaker 3**	**All speakers**
*Utterances*	15	14	15	44
*Total Frames (percentage of frames in relation with the total for all speakers)*	836 (53.2%)	283 (18.0%)	453 (28.8%)	1572
*Nasal frames (percentage of nasal frames) / Non-nasal frames (percentage of non-nasal frames)*	357 (42.7%) / 479 (57.3%)	195 (68.9%) / 88 (31.1%)	249 (55.0%) / 204 (45.0%)	801 (51.0%) / 771 (49.0%)
*Total Zones (percentage of nasal and non-nasal zones in relation with the total for all speakers)*	76 (35.3%)	65 (30.2%)	74 (34.4%)	215
*Nasal Zones (percentage of nasal zones) / Non-nasal Zones (percentage of non-nasal zones)*	45 (59.2%) / 31 (40.8%)	45 (69.2%) / 20 (30.8%)	45 (60.8%) / 29 (39.2%)	135 (62.8%) / 80 (37.2%)

The first row presents the number of utterances per speaker and the total number of utterances for all speakers. The second row presents the total frames: the percentages were calculated in relation to the total amount of frames of all speakers (e.g. Speaker 3 data contains 28.8% of the frames found in this corpus). The third row presents the number of nasal and non-nasal frames and the percentage values concern the distribution of the frame type (e.g. 42.7% of the frames for Speaker 1 are nasal). In the third and fourth rows we apply the same presentation structure (as in the second and third rows) but concerning nasal and non-nasal zones.

**Fig 12:**
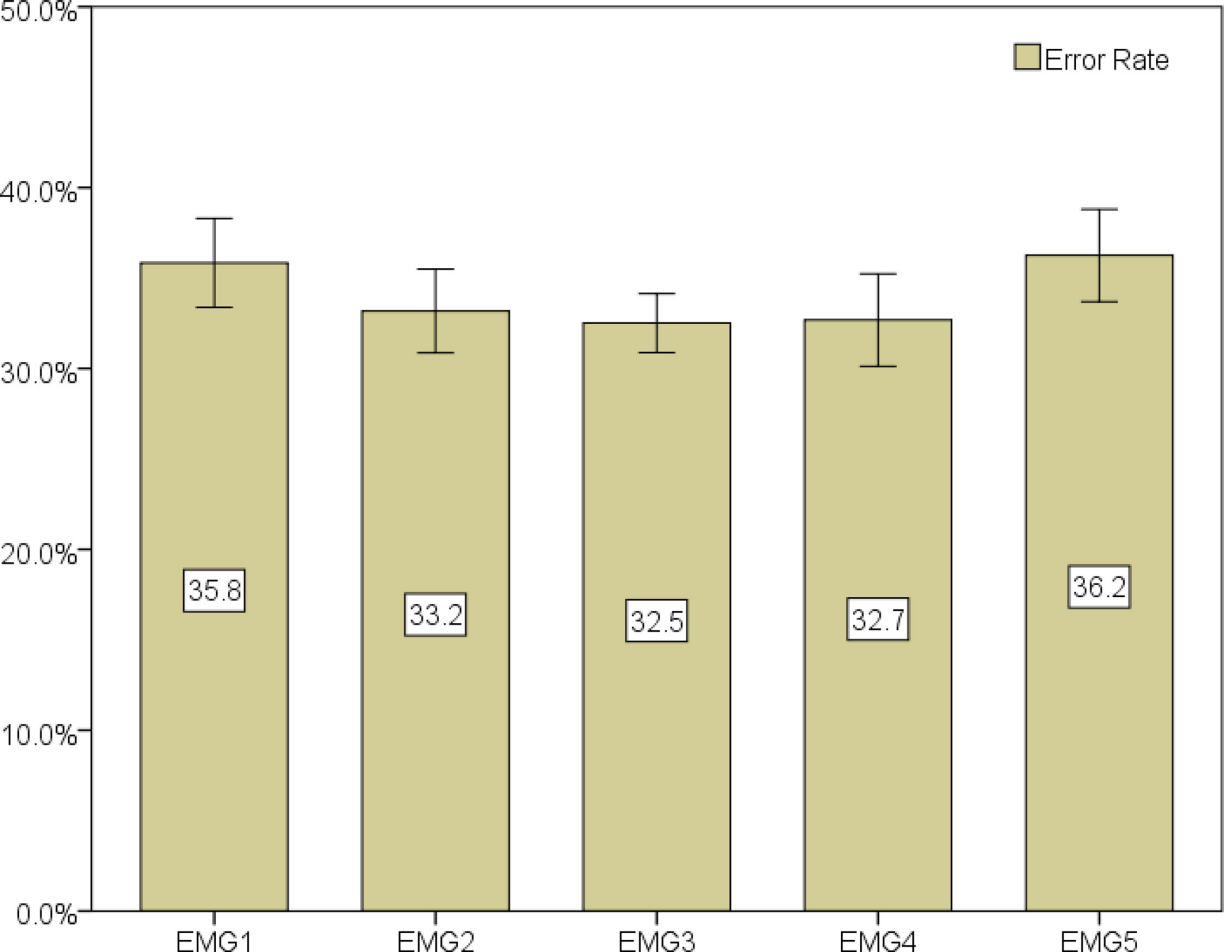
Classification results (mean value of the 10-fold for error rate, sensitivity and specificity) for all channels and all speakers. Error bars show a 95% confidence interval.

Classification was initially performed using the data from all speakers. The error rate results are depicted in Fig 12 and the sensitivity and specificity results are presented in Table 2. Besides the mean value of the 10-fold cross validation, 95% confidence intervals are also included. Results show a best result for EMG Channel 3 with a 32.5% mean error rate, presenting a mean sensitivity and mean specificity of 65.5% and 69.4%, respectively. Channels 4 and 2 presented similar results and achieved second and third best results with mean error rates of 32.7% and 33.2% and with slightly lower sensitivity values of 61.3% and 63.0%, and higher specificity values of 73.0% and 70.4%.

**Table 2:** Mean sensitivity and specificity measures (%) for each EMG channel with a 95% confidence interval.

**EMG channel**	*Specificity*	*Sensitivity*
*1*	67.1±3.2	61.1±3.8
*2*	70.4±2.5	63.0±2.9
*3*	69.4±2.7	65.5±3.3
*4*	73.0±2.1	61.3±4.3
*5*	67.5±3.6	59.8±3.1

**Fig 13:**
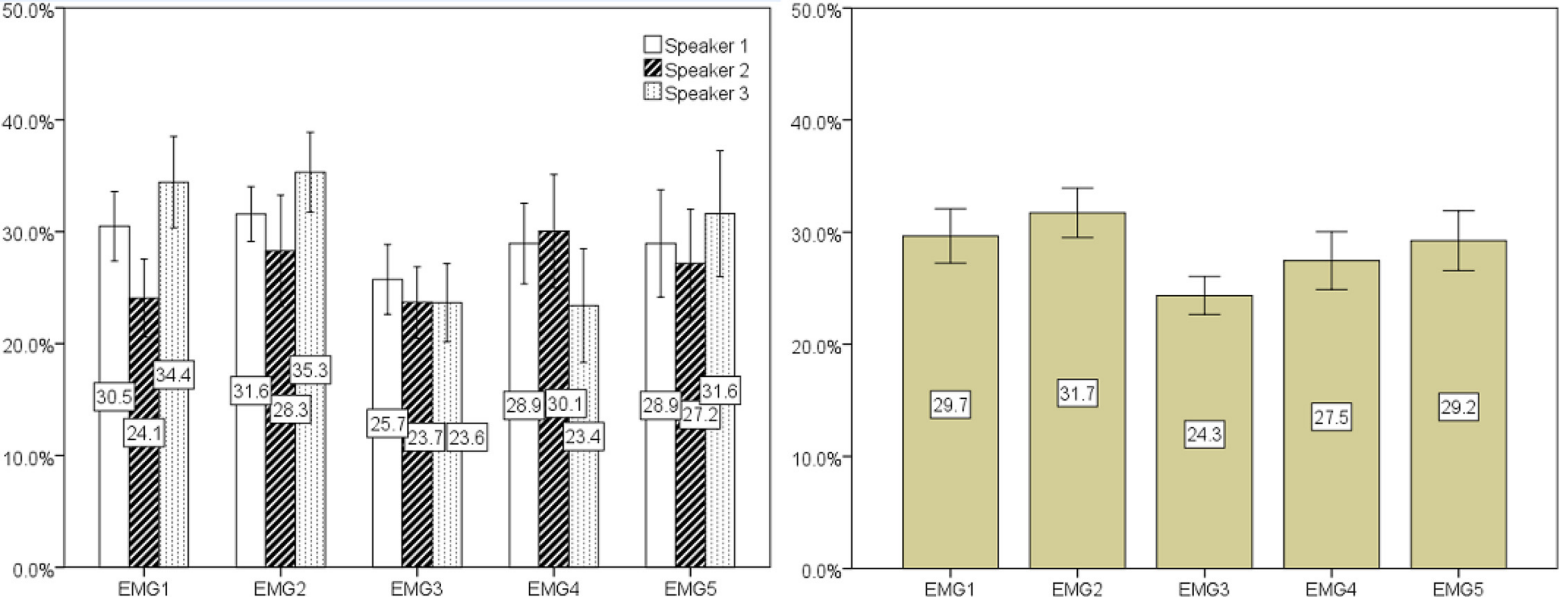
The graph on the left shows the mean error rate for each speaker clustered by EMG channel. The graph on the right shows the mean of the error rates from each speaker also clustered by EMG channel. Error bars show a 95% confidence interval.

Classification was also performed for each individual speaker. Error rates are shown in Fig 13, for each speaker (left) and overall, per channel (right), along with the corresponding 95% confidence interval. The best overall result of 24.3% was attained using EMG channel 3. The best results for each individual speaker were found for speaker 3 with 23.4% and 23.6% mean error rate in EMG channel 4 and 3. For speaker 1 and 2, EMG channel 3 presented the best results with 25.7% and 23.7% mean error rate. On average, speaker 1 presented the least variability of results, as shown by the confidence intervals. It is also interesting to note the difference of results in EMG channel 1, where speaker 2 attained a mean error rate of 24.1%. However, the class distribution slightly changes for speaker 2 with 68.9% nasal frames, compared with 42.7% and 55.0% frames for speaker 1 and 3. A closer look into the data of speaker 2 also reveals that the higher amount of nasal frames is explained by breaths between words, which implies opening the velum.

**Fig 14:**
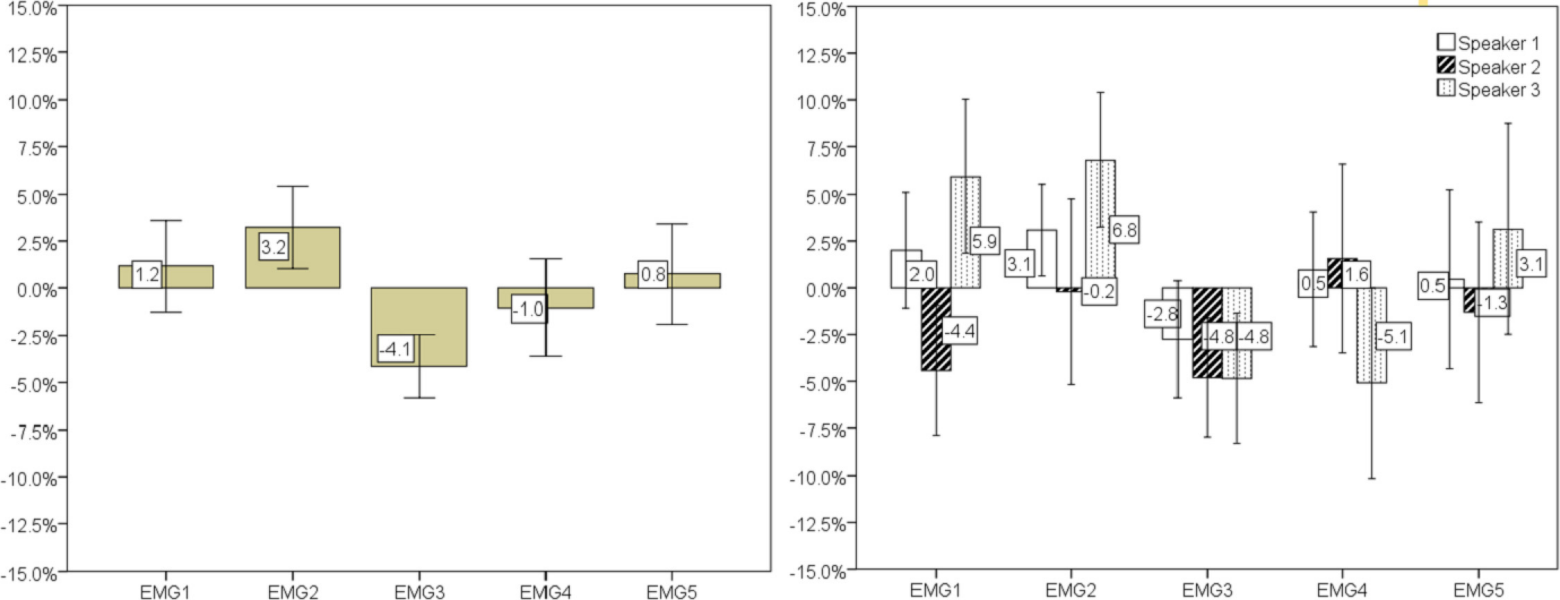
Difference between the mean error rate of all channels and the respective result of each channel for all (left) and each (right) speaker. Error bars show a 95% confidence interval.

Looking at the results from a different perspective, if we subtract the global mean error rate of all channels the following results were found. As depicted in Fig 14, Channel 3 exhibited a mean error rate 4.1% below the global mean error rate of all channels. Analyzing it by speaker, the best result was achieved for EMG channel 4 of speaker 3 with 5.1% below the mean. A noticeable result was also found for EMG channel 1 of speaker 2, obtaining results below the mean error rate. However, for these speakers, the 95% confidence interval was considerably higher, showing some instability in the results.

When looking at the results, grouped by nasal vowel, as shown in Table 3, an improvement can be noticed, particularly for the [u~] case, with a 27.5% mean error rate using EMG channel 3.

**Table 3:** Mean error rate grouped by nasal vowel.

**EMG Channel**	[6~p6] [p6~p6] [p6~]	[e~p6] [pe~p6] [pe~]	[i~p6] [pi~p6] [pi~]	[o~p6] [po~p6] [po~]	[u~p6] [pu~p6] [pu~]
*1*	36.2%	33.6%	38.7%	**32.9%**	35.6%
*2*	**34.2%**	33.9%	34.6%	**33.5%**	29.9%
*3*	39.8%	31.4%	35.8%	**29.4%**	**27.5%**
*4*	38.8%	**28.6%**	**32.8%**	35.1%	28.1%
*5*	39.5%	36.8%	36.1%	33.5%	35.0%
*Mean*	37.7%	32.9%	35.6%	32.9%	31.2%

To assess if any advantage could be extracted from using channel combination, to improve classification, we have also experienced classification with multiple EMG channels. The most relevant combinations are shown in Table 4. The best results for all speakers and for each speaker individually were worse than the ones obtained previously.

**Table 4:** Mean error rate using multiple channels combinations.

**EMG Channel**	**All speakers**	**Speaker 1**	**Speaker 2**	**Speaker 3**
*1 + 3*	35.0%	30.1%	**24.7%**	29.5%
*2 + 3*	36.3%	**28.0%**	31.3%	33.9%
*2 + 4*	34.9%	31.4%	30.2%	33.0%
*3 + 4*	**32.9%**	28.7%	33.0%	**27.6%**
*2 + 3 + 4*	35.7%	29.0%	33.5%	32.1%
*1 + 3 + 4 + 5*	39.1%	36.3%	32.5%	34.9%

#### Statistical Analysis

To assess significant differences among the EMG channels’ error rate, we performed a repeated-measures ANOVA considering a pair-wise comparison between EMG channels. The results, summarized in Table 5, show as significant (p < 0.05) the differences between EMG channel 3 and the remaining channels and between channels 2 and 4. Regarding the other channel pairs no significant differences were found. For some EMG channel pairs, statistically significant differences were found among speakers. In this analysis, no serious violations of normality and homogeneity of variance were found, and sphericity was guaranteed.

**Table 5:** Results of the repeated-measures ANOVA analysis for the EMG channel pairs that attained significance level.

**EMG Channel Pair**	**F(1, 27)**	**p-Value**	**Speaker Effect**
*3 vs 1*	16.112	p<0.001	Significant
*3 vs 2*	27.532	p<0.001	Significant
*3 vs 4*	6.603	p = 0.016	Non significant
*3 vs 5*	16.008	p<0.001	Non significant
*2 vs 4*	7.394	p = 0.011	Significant

### Nasal and Non-nasal Zone Classification

The information extracted from the RT-MRI signal, although not possible to use in a real classification scenario, allows further exploring and validating of our methodology. As described in section 0, the RT-MRI information allowed us to split the EMG signal into nasal and non-nasal zones. Thus, by knowing the zone boundaries in the EMG signal, we could conduct a classification experiment based on the majority of nasal/non-nasal frames of a certain zone. Assuming a decision by majority, a zone is nasal if the number of nasal frames is equal or higher than the number of non-nasal frames. Results using this technique are depicted in Table 6. These results showed an absolute improvement of 11.0% when compared to what was achieved earlier using a single frame of the signal. When looking at a specific part of the zone, the results were still better than the ones achieved previously. The most important information seemed to be located in the initial half of each zone, since an accuracy degradation trend was observed for all channels when the initial part of the zone was not considered. When considering only the nasal zones, the error rate reached 12.6% for EMG channel 4 and the same trend of better results in the initial part of the zone was also verified, as depicted in Table 7. When taking into account only the non-nasal zones we observed the best results in EMG channel 3, using only the central part of the non-nasal zones (i.e. the 25%-75% interval) with 25.0% mean error rate.

**Table 6:** Mean error rates using a classification technique based on the majority of nasal/non-nasal frames for each zone.

	**Part of the zone considered**			
**EMG Channel**	*[0–100%]*	*[0%-50%]*	*[25%-75%]*	*[50%-100%]*
*1*	25.2%	28.0%	24.3%	**27.7%**
*2*	**21.5%**	25.2%	28.7%	32.2%
*3*	25.7%	27.1%	28.7%	33.7%
*4*	24.3%	**22.9%**	**23.3%**	31.7%
*5*	32.2%	29.0%	30.7%	36.1%
*Mean*	**25.8%**	26.5%	27.1%	32.3%

Each column of the table shows which part of the zone is being considered, where 0% represents the zone start and 100% the zone end (e.g. in 50%-100% interval only the samples in the last half of each zone are being considered).

**Table 7:** Mean error rates using a classification technique based on the majority of nasal/non-nasal frames for each nasal zone.

	**Part of the NASAL zone considered**			
**EMG Channel**	*[0–100%]*	*[0%-50%]*	*[25%-75%]*	*[50%-100%]*
*1*	18.5%	18.5%	20.5%	22.8%
*2*	14.1%	19.3%	26.0%	22.8%
*3*	21.5%	23.7%	29.9%	26.0%
*4*	**12.6%**	**15.6%**	**13.4%**	**16.5%**
*5*	23.7%	24.4%	22.1%	27.6%
*Mean*	**18.1%**	20.3%	22.4%	23.2%

### Reproducibility Assessment

The EMG signal varies across recording sessions, even for the same speaker. For that reason, we assessed the effect of such variability in our study. Using 4 additional sessions from speaker 1, recorded at different times, we have conducted the same analysis as presented in the previous sections. The results for all sessions were very similar, showing evidence that the existing variability among sessions had no major influence on the results.

**Fig 15:**
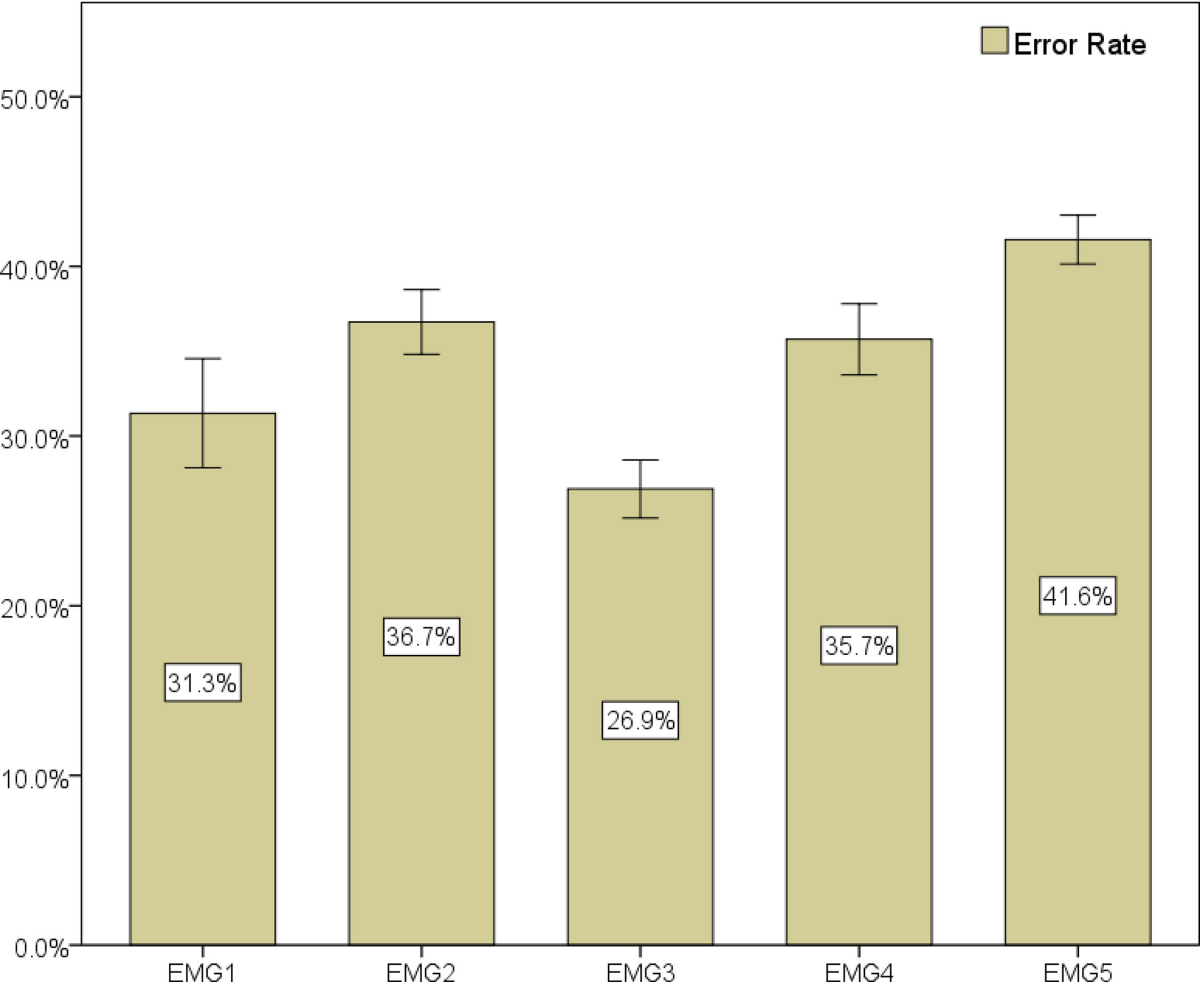
Classification results (mean value of the 10-fold for error rate, sensitivity and specificity) for all channels of speaker 1. These results are based on four additional sessions from this speaker recorded a posteriori. Error bars show a 95% confidence interval.

Considering a distribution of 2448 total frames, where 47.0% are nasal and 53.0% are non-nasal, we observe a best error rate of 26.9% for EMG channel 3, as depicted in Fig 15. Sensitivity and specificity results for these additional sessions can be found in Table 8: channel 3 presents the highest values with 76.8% and 69.0% respectively.

**Table 8:** Mean sensitivity and specificity measures (%) with a 95% confidence interval for each EMG channel of speaker 1.

**EMG channel**	*Specificity*	*Sensitivity*
*1*	61.6±4.5	71.9±1.7
*2*	61.2±3.5	65.1±3.4
*3*	69.0±2.0	76.8±1.5
*4*	59.5±3.1	68.6±2.6
*5*	55.7±3.4	60.8±2.0

These results are based on four additional sessions from this speaker recorded a posteriori.

Taking into consideration that the dataset distribution presents a higher amount of nasal frames, if we compare the results with the ones reported in the previous sections, a slightly higher error rate can be noticed, particularly for channels 4 and 5. For EMG channel 3, the best in both conditions, the difference is very small (1.2% absolute value).

## Discussion

The global results of this study point to the fact that the selected approach can be used to reduce the error rate caused by nasality in languages such as EP where this characteristic is particularly relevant. Looking at the results of our analysis, there is a noticeable trend that points to the electrode pairs placed below the ear between the mastoid process and the mandible in the upper neck area as being the sensors with less crosstalk and as being the most promising for detecting the myoelectric signal generated by the velum movement. In a first stage, when overlaying the aligned RT-MRI and EMG signals the obtained matching is more evident for channels 2, 3 and 4, particularly for nasal vowels in medial and final word positions. However, when looking at the close vowel case and at the vowels in an isolated context, EMG channels 3 and 4 emerge as the most likely. In the isolated context, channel 1 also presents interesting results, showing that a more accurate filtering might be necessary to obtain the desired signals from this channel. In the case of EMG channel 5, a signal matching the expected tongue movement can be noticed, making it unclear if velar information is actually being captured. Still in the visual analysis, the fact that a more evident matching is obtained for the medial and final word position suggests that it would be interesting to further analyze the differences between nasal vowels positions. The word-final context only requires an opening movement, while a word-initial position could conceivably in some cases require only a closing movement. At least one could say that any opening movement is under weak temporal constraints. The word-internal context requires a clear opening-closing movement under strict temporal constraints given by the linguistically appropriate duration between the flanking plosives. Thus it is perhaps not surprising that the latter context gives clear results. However, different contexts, ideally where no jaw movement is required, should be considered to discard or minimize eventual muscle crosstalk.

In our data analysis, using a different approach, we have also compared the information present in the nasal zones of the EMG signal with the velar information of the RT-MRI signal using mutual information to measure the relation between the signals. The higher results were found in EMG channels 3, 4 and 5, being aligned with our visual analysis of the signal.

The overall results seem to indicate that, for these speakers, velum information is actually being captured by the EMG sensors, however, it is not clear which muscles are being measured. A more detailed discussion about the classification results and the reproducibility of this study is presented in the following subsections.

### Classification

Following our data analysis we investigated if it was possible to develop a classifier that enabled us to distinguish between nasal and non-nasal frames of an EMG signal in a realistic frame independent scenario. The results for this scenario showed that it is possible to distinguish between the two frame classes, following the results from our previous analysis, particularly in EMG channels 2, 3 and 4. Specificity and sensitivity measures found for these channels are also slightly higher when compared with the remaining channels, showing that more positive/negative results were accurately found. When looking at the results of the classifier per speaker an improvement in the results can be noticed, showing a better modelling of our classification task. In this case, EMG channel 3 presents the best results for all speakers. EMG channel 1 and 4 present substantial differences between speakers and recording sessions, showing that further exploration is required, particularly for channel 1, which, due to its position near several bone structures, may present high sensitivity regarding sensor position or anatomic structure in that area for tracking this set of muscles.

The statistical analysis also shows significant differences can be found between the results of EMG channel 3 and the remaining EMG channels, in line with what was found above, where EMG channel 3 emerges as the channel with the best results.

In our study, we have also attempted to combine multiple channels, however, this did not improve the obtained results. This can be an indication of the following: (1) the muscles’ signals captured by the EMG channels overlap, thus, it does not improve the nasal/non-nasal class separation; (2) due to the superimposition of muscles in that area, adding a new channel with information of other muscles will create a less accurate model of the classes; (3) the muscles related with velum movement are not being captured by the added channel(s).

In a scenario where each zone is classified based on the majority frame type (nasal or non-nasal), we find a noteworthy improvement in the accuracy rates, reaching 12.6% for all users. Although this is not a realistic scenario in the sense that an interface using surface EMG to detect velum movement would not know *a priori* the nasal zone boundary, these results allow us to understand that the use of neighboring frames and introducing frame context might help to improve the accuracy of our methodology. It is also interesting to note the higher error rate in non-nasal frames and the fact that better results are obtained not using the whole zone but only the central part of each zone.

An alternative approach to the developed classifier would be to detect velum movement events, since EMG activity is most likely to be found when the position of the velum needs to be changed. However, based on the results obtained in this study and the physiology of the area in question, it does not seem likely that a position without other muscle crosstalk can be found. As such, an event-based approach would need to find a way to avoid false positives originating from neighboring muscles.

### Reproducibility

One aspect that deserves attention concerns how inter- and intra-speaker variability might influence the gathered results. Regarding inter-speaker variability, the first issue to discuss is if the number of speakers is adequate. In this regard, we argue that, for the purpose of this exploratory study, three speakers encompass enough variability because of the following:: 1) They yield some anatomical and physiological variation that, to some extent, influence how sensors are placed, considering the landmarks and criteria we describe in section 2.4.1; 2) Since nothing was imposed on this matter, the speakers have different/varying speech rates, which introduce variability in the EMG-MRI alignment; and 3) the corpus includes nasals in different word positions and contexts, produced along one hour recording sessions, providing a large amount of nasality data per speaker. Considering these variability factors, and the results obtained for the error rates, similar among speakers, we believe that inter-speaker variability is reasonably addressed. Nevertheless, a larger number of speakers would be desirable, but a very risky option at this time, considering that there was no source in the literature to support the viability of this study and RT-MRI acquisitions are very expensive. Note that we do not intend to generalize the interpretation of the results presented in this study. The well know variability across speakers, found in EMG signals [39,40], and the number of speakers involved, precludes such conclusion. The extent to which the EMG is able to capture velum information may vary among speakers. Nevertheless, the results gathered provide enough evidence that it is possible to do it, and contribute with a set of EMG sensor positions. This allows moving to the next stage and pursue the research with a larger number of speakers. In terms of classification results we noticed a stable trend for EMG channel 3. Regarding the other channels, the differences in terms of classification results may be explained by the physiological differences between speakers. For example, the EMG channel 1 was placed in an area near to several structures (e.g. the inferior maxillary bone) that may interfere with the signal from deeper muscles. Thus, for these channels it is not possible to draw a stable conclusion.

On the subject of intra-speaker variability, additional sessions from a random speaker recorded a posteriori, support the previously achieved results, further evidencing that, for the speaker in question, the position selected for EMG channel 3 attains the best results. In that sense, intra-speaker variability, sometimes found in EMG sensors placed in the regions of the face and neck [40], did not have a strong impact on the results for this speaker. The fact that it was always the same experienced person placing the EMG sensors, using the same references, and the same hardware, may have contributed to lower the variability between recording sessions of the same speaker.

## Conclusions and Future work

The work here presented used two distinct sources of information–surface EMG and RT-MRI–in order to address the challenge of nasality detection in EMG-based silent speech interfaces. The information extracted from the RT-MRI images allowed us to know when to expect nasal information. Thus, by synchronizing both signals, based on simultaneously recorded audio signals from the same speaker, we were able to explore the existence of useful information in the EMG signal about the velum movement. The positioning of the surface EMG electrode was based on probable locations for detecting the targeted muscles.

Our results point to the possibility of developing an SSI based on surface EMG for EP that detects the muscles associated with the movement of the velum, as well as providing a background for future studies in terms of sensor positioning. The results of this study show that in a real use situations error rates of 23.7% can be achieved for sensors positioned below the ear between the mastoid process and the mandible in the upper neck region, and that careful articulation and positioning of the sensors may influence nasal vowel detection results.

The presented outcomes indicate that the described approach can be a valuable contribution to the state-of-the-art in the area of EMG speech interfaces, and that it can be applied in parallel with other techniques. Additionally, although the methodology used in this study partially relies on RT-MRI information for scientific substantiation, a technology which requires a complex and expensive setup, the proposed solution to detect nasality is solely based on a single, non-invasive sensor of surface EMG. Thus, the development of an SSI based on EMG for EP, with language adapted sensor positioning, now seems to be a possibility.

For future work, we would like to extend this study by including more speakers and nasal consonants, which was not possible due to the lack of RT-MRI information for that case. We also intend to analyze other non-invasive approaches that may be able to detect nasality, to be used in parallel with surface EMG, such as ultrasonic Doppler sensing [41]. Furthermore, a solution for an accurate and replicable positioning of surface EMG sensors needs to be considered. We would also like to further explore the differences in the resulting EMG patterns of the three nasal vowels’ position (i.e. nasal vowel in initial, internal and final position) and to consider an event-based classification model, which would detect velum movement.

## Supporting Information

S1 Data Zip file with the RT-MRI velum information, surface EMG data for the five channels and the synchronized signals of all speakers. (ZIP)
